# Supplementary material for: Development and Piloting of a Web-Based Tool to Teach Relative and Absolute Risk Reductions
Source: Int J Environ Res Public Health. 2022 Dec 1;19(23):16086. doi: 10.3390/ijerph192316086 (PMC9739880; doi:10.3390/ijerph192316086)
Supplement: Supplementary file 1 [file ijerph-19-16086-s001.zip › Table S1. Description of the systematic search strategy.pdf]

Table S1. Description of the systematic search strategy

<table>
<tr><td colspan="2">Systematic literature search</td></tr>
<tr><td>Inclusion criteria</td><td>Exclusion criteria</td></tr>
<tr><td><u>Population:</u> Laypeople, healthcare professionals
<u>Intervention:</u> Web-based learning material, for teaching relative and absolute risk reduction
<u>Control</u>: Not defined
<u>Outcome:</u> Not defined
<u>Study design:</u> Randomised controlled trials, systematic reviews, pilot studies, feasibility studies</td><td><u>Intervention:</u> Type of material: Books available online, Video recordings of lectures, Podcasts Studies were excluded if information on the educational material was missing or access restrictions prevented analysis.</td></tr>
<tr><td colspan="2">Note: Information on the content of the educational intervention was requested from study authors for articles published up to 2014. This was done for three articles, of which all three remained unanswered. Paid access to materials was purchased.</td></tr>
<tr><td colspan="2">Search strategies</td></tr>
<tr><td colspan="2">Note: The systematic literature search followed a two-stage process for systematic reviews and randomized controlled trials (RCT) (Search 1) and pilot and feasibility studies (Search 2).</td></tr>
<tr><td colspan="2">1. Medline via PubMed (RCT)</td></tr>
<tr><td>S1</td><td>Education [Title/Abstract] OR instruction [Title/Abstract] OR learning material [Title/Abstract] OR teaching material [Title/Abstract] OR learning tool [Title/Abstract] OR toolkit [Title/Abstract] OR training [Title/Abstract] OR e-learning [Title/Abstract] OR "Education"[Mesh] OR "education" [Subheading] OR "Teaching"[Mesh] OR "Teaching Materials"[Mesh] OR "Education, Distance"[Mesh] OR "Mobile Applications"[Mesh] OR "Education, Medical"[Mesh] OR "Health Education"[Mesh] OR "Education [Title/Abstract] OR Nursing"[Mesh]</td></tr>
<tr><td>S2</td><td>Statistics [Title/Abstract] OR relative risk [Title/Abstract] OR absolute risk [Title/Abstract] OR relative effects [Title/Abstract] OR absolute effects [Title/Abstract] OR critical appraisal [Title/Abstract] OR Health literacy [Title/Abstract] OR Evidence based nursing [Title/Abstract] OR Evidence based medicine [Title/Abstract] OR Evidence based practice [Title/Abstract] OR "Statistics as Topic/education"[Mesh] OR "Data Interpretation OR Statistical/methods"[Mesh] OR "Data Interpretation Statistical/standards"[Mesh] OR "Evidence-Based Nursing/education"[Mesh] OR "Evidence-Based Medicine/education"[Mesh] OR "Evidence-Based Practice/education"[Mesh] OR "Information Literacy"[Mesh] OR "Health Literacy"[Mesh]</td></tr>
<tr><td>S3</td><td>S1 AND S2</td></tr>
<tr><td colspan="2">Filter: Systematic Reviews; RCT</td></tr>
<tr><td colspan="2">2. Medline via PubMed (pilot and feasibility studies)</td></tr>
<tr><td>S1</td><td>Education [Title/Abstract] OR instruction [Title/Abstract] OR learning material [Title/Abstract] OR teaching material [Title/Abstract] OR learning tool [Title/Abstract] OR toolkit [Title/Abstract] OR training [Title/Abstract] OR e-learning [Title/Abstract] OR "Education"[Mesh] OR "education" [Subheading] OR "Teaching"[Mesh] OR "Teaching Materials"[Mesh] OR "Education, Distance"[Mesh] OR "Mobile Applications"[Mesh] OR "Education, Medical"[Mesh] OR "Health Education"[Mesh] OR "Education [Title/Abstract] OR Nursing"[Mesh]</td></tr>
<tr><td>S2</td><td>Statistics [Title/Abstract] OR relative risk [Title/Abstract] OR absolute risk [Title/Abstract] OR relative effects [Title/Abstract] OR absolute effects [Title/Abstract] OR critical appraisal [Title/Abstract] OR Health literacy [Title/Abstract] OR Evidence based nursing [Title/Abstract] OR Evidence based medicine [Title/Abstract] OR Evidence based practice [Title/Abstract] OR "Statistics as Topic/education"[Mesh] OR "Data Interpretation OR Statistical/methods"[Mesh] OR "Data Interpretation Statistical/standards"[Mesh] OR "Evidence-Based Nursing/education"[Mesh] OR "Evidence-Based Medicine/education"[Mesh] OR "Evidence-Based Practice/education"[Mesh] OR "Information Literacy"[Mesh] OR "Health Literacy"[Mesh]</td></tr>
<tr><td>S3</td><td>Pilot [Title/Abstract] OR feasibility [Title/Abstract]</td></tr>
<tr><td>S4</td><td>S1 AND S2 AND S3</td></tr>
</table>

Table S1. Continued

**3. PsycInfo (Systematic Reviews)**	
S1	(AB) Education OR (AB) instruction OR (AB) learning materials OR (AB) teaching materials OR (AB) learning tools OR (AB) toolkit OR (AB) training OR (AB) e-learning OR (AB) web tools OR DE "Education" OR DE "Electronic Learning" OR DE "Mobile Applications" OR DE "Computer Applications"
S2	(AB) Statistics OR (AB) relative risk OR (AB) absolute risk OR (AB) relative effects OR (AB) absolute effects OR (AB) Critical Appraisal OR (AB) Health Literacy OR (AB) Evidence based nursing OR (AB) Evidence based medicine OR (AB) Evidence based practice OR DE "Evidence Based Practice" OR DE "Health Literacy"
S3	S1 AND S2
Filter: Systematic Reviews; Meta analyses, Literature Review	
**4. PsycInfo (RCT)**	
S1	(AB) Education OR (AB) instruction OR (AB) learning materials OR (AB) teaching materials OR (AB) learning tools OR (AB) toolkit OR (AB) training OR (AB) e-learning OR (AB) web tools OR DE "Education" OR DE "Electronic Learning" OR DE "Mobile Applications" OR DE "Computer Applications"
S2	(AB) Statistics OR (AB) relative risk OR (AB) absolute risk OR (AB) relative effects OR (AB) absolute effects OR (AB) Critical Appraisal OR (AB) Health Literacy OR (AB) Evidence based nursing OR (AB) Evidence based medicine OR (AB) Evidence based practice OR DE "Evidence Based Practice" OR DE "Health Literacy"
S3	(AB) rct OR (AB) randomized controlled trials OR (AB) randomized control trial OR (AB) randomised controlled trial
S4	S1 AND S2 AND S3
**5. PsycInfo (pilot and feasibility studies)**	
S1	(AB) Education OR (AB) instruction OR (AB) learning materials OR (AB) teaching materials OR (AB) learning tools OR (AB) toolkit OR (AB) training OR (AB) e-learning OR (AB) web tools OR DE "Education" OR DE "Electronic Learning" OR DE "Mobile Applications" OR DE "Computer Applications"
S2	(AB) Statistics OR (AB) relative risk OR (AB) absolute risk OR (AB) relative effects OR (AB) absolute effects OR (AB) Critical Appraisal OR (AB) Health Literacy OR (AB) Evidence based nursing OR (AB) Evidence based medicine OR (AB) Evidence based practice OR DE "Evidence Based Practice" OR DE "Health Literacy"
S3	(AB) pilot OR (AB) feasibility
S4	S1 AND S2 AND S3
**6. Psyndex via PubPsych (Systematic Reviews, RCT, pilot and feasibility studies)**	
S1	Education OR learning material OR learning tool OR toolkit OR e-learning OR Webtool OR Unterricht
S2	Statistics OR relative risk OR absolute risk OR Critical Appraisal OR Health Literacy OR Therapieeffekt
S3	S1 AND S2
Filter: DatabasePsyndex	

Internet search

**Inclusion criteria**	**Exclusion criteria**
Target group: Laypeople, healthcare professionals<br>Educational intervention: Web-based learning material, for teaching relative and absolute risk reduction	Educational intervention: Books available online, Video recordings of lectures, Podcasts

Search strategies

Internet searches were conducted via Google and learning resource databases from Teachers of Evidence-Based Health Care, Merlot and National Collaborating Centre for Methods and Tools. The browser settings were reset for the "Google search" and the private mode was used. Search terms were used in German and English. For each search term, the first ten pages were examined.

**Resource**	**Search terms / Filter**
**1. Teachers of Evidence-Based Health Care**	Filter: Key Concept 2-3b “Relative Measures Of Effects Can Be Misleading”

Table S1. Continued

**2. Merlot**	Treatment claims, relative risk reduction, absolute risk reduction, statistics<br>Category: Science and Technology<br>Subcategory: Health Sciences
**3. National Collaborating Centre for Methods and Tools**	Treatment claims, relative risk reduction, absolute risk reduction, relative risk, absolute risk, statistics
**4. Google**	Relative risk reduction education, relative risk reduction, relative Risikoreduktion, absolute risk reduction education, absolute risk reduction, absolute Risikoreduktion
