# Supplementary material for: Development and Piloting of a Web-Based Tool to Teach Relative and Absolute Risk Reductions
Source: Int J Environ Res Public Health. 2022 Dec 1;19(23):16086. doi: 10.3390/ijerph192316086 (PMC9739880; doi:10.3390/ijerph192316086)
Supplement: Supplementary file 1 [file ijerph-19-16086-s001.zip › Table S2. Identified educational interventions through the systematic literature review and internet search.pdf]

Table S2. Identified educational interventions through the systematic literature review and internet search

Author (Year)/Titel	Source	Link
**Anger & Steilmann, (2016)**<br>Entwicklung und Pilotierung einer audiovisuellen Patienteninformation zur Risikoreduktion für Menschen mit Multipler Sklerose	Literature review	
**Coppus et al., EU-EBM Projekt (2007)**<br>A clinically integrated curriculum in evidence-based medicine for just-in-time learning through on-the-job training	Literature review	
**Ilic et al., (2013)**<br>Adopting a blended learning approach to teaching evidence based medicine: A mixed methods study	Literature review	
**Attia, (2018)**<br>Studying Studies: Part I – relative risk vs. absolute risk	Google	https://peterattiamd.com/ns001/
**Barratt et al., (2004)**<br>Tips for learners of evidence-based medicine: 1. Relative risk reduction, absolute risk reduction and number needed to treat	Google	http://www.cmaj.ca/content/171/4/353.full
**Breastcancer.org, (2019)**<br>Examples of Risk Increasing and Decreasing	Google	https://www.breastcancer.org/risk/understand/examples
**Buckingham, Fisher, & Saunders, (2008)**<br>Statistical Calculations Interpreting the Importance and Precision of Therapeutic Results	Google	http://www.ebm.med.ualberta.ca/TherapyCalc.html
**Cates, (2005)**<br>Understanding statistics 1: presenting data from clinical trials	Google	https://learning.bmj.com/learning/module-intro/understanding-statistics.html?moduleId=5003158
**Gnadt & Dircks, (2016)**<br>OR, RR, NNT<br>Grundlagen der medizinischen Statistik: Klinische Studien und ihre Bewertung,<br>Teil 3	Google	https://www.deutsche-apotheker-zeitung.de/daz-az/2016/daz-5-2016/or-rr-nnt
**HealthNewsReview.org, (2019)**<br>Reporting the findings: Absolute vs. relative risk.	Testing Treatments	https://www.healthnewsreview.org/toolkit/tips-for-understanding-studies/absolute-vs-relative-risk/

Table S4. Continued

**medistat, (2019)**<br>Absolute und relative Risikoreduktion	Google	https://www.medistat.de/glossar/analyse-von-haeufigkeiten/relative-risikoreduktion/
**Molina, (2018)**<br>King of Kings	Google	https://www.cienciasinseso.com/en/tag/relative-risk-reduction/
**National Breast Cancer Coalition, (2013)**<br>Understanding Absolute Risk Reduction Versus Relative Risk Reduction	Google	http://www.breastcancerdeadline2020.org/breast-cancer-information/quality-care-guide/choice/making-decisions-about-your-care/understanding-absolute-risk.html
**National Collaborating Centre for Methods and Tools, (2019)**<br>What's the Risk? Understanding Absolute and Relative Risk Reduction	National Collaborating Centre for Methods and Tools	https://www.nccmt.ca/capacity-development/videos?v=548#ure1
**Neye, (2004)**<br>Wie gross ist der wirkliche Nutzen für Patienten?	Google	https://www.pharmazeutische-zeitung.de/inhalt-09-2004/pharm3-09-2004/
**O'Rourke, (1998)**<br>Relative Risk and Absolute Risk Interpretation	Google	http://www.uvm.edu/~dhowell/gradstat/psych340/Labs/risk.html#RRR
**Public Health Action Support Team, (2017)**<br>Summarising binary data	National Collaborating Centre for Methods and Tools	https://www.healthknowledge.org.uk/e-learning/statistical-methods/practitioners/summarising-binary-data
**Sambunjak D, Cumpston M, Watts C., (2017)**<br>Module 6: Analysing the data	National Collaborating Centre for Methods and Tools	https://training.cochrane.org/interactivelearning/module-6-analysing-data.
**Tape, (not specified)**<br>Measures of Effect Size of an Intervention	Google	http://gim.unmc.edu/dxtests/effect1.htm
**Therapeutics Initiative, (1996)**<br>Evidence Based Drug Therapy<br>What Do the Numbers Mean?	Google	https://www.ti.ubc.ca/pages/letter15.htm
**Universität Hamburg, (2018)**<br>Risikoreduktion<br>Risikoreduktion besser einschätzen	Google	https://www.gesundheit.uni-hamburg.de/wissen/grundwissen/risikoreduktion.html
**University of Massachusetts Medical School, (not specified)**<br>Relative risk, absolute risk and number-needed-to-treat	Testing Treatments	https://onlinetraining.umassmed.edu/ay1213011
