# Supplementary material for: Development and Piloting of a Web-Based Tool to Teach Relative and Absolute Risk Reductions
Source: Int J Environ Res Public Health. 2022 Dec 1;19(23):16086. doi: 10.3390/ijerph192316086 (PMC9739880; doi:10.3390/ijerph192316086)
Supplement: Supplementary file 1 [file ijerph-19-16086-s001.zip › Table S3. Category system.pdf]

Table S3. Category system

Main category	Subcategory	Definition	Anchor example
1. Acceptance<br><br>Acceptance includes the satisfaction of all participants with the tool as well as the emotional reactions and all statement concerning the relevance and hypothetical benefit and recommendation of the tool.	1.1 Satisfaction	All text passages are included from which a statement concerning the satisfaction of the participants with the tool can be derived.	*"Yes, no it's nice to be a little more based in one's assessment."*
	1.2 Emotional reaction	All text passages are included from which a statement from the participants can be derived concerning a reaction such as pleasure, anger or fear, for example	*"And when I read this through, it's always impressive how blinded one actually becomes about some things."*
	1.3 Relevance	All text passages are included in which the participants mention something about the relevance of the content and/or the practical application of the tool.	*"I do think that it is very relevant having to think about just how such events should be interpreted."*
	1.4 Benefit/ Recommendation	All text passages are included in which the participants address the hypothetical benefit or recommendation to acquaintances, relatives or colleagues.	*"I really would recommend the tool to others so that more people deal with these issues."*
2. Applicability<br><br>Applicability includes statements concerning structure, handling and design of the tool	2.1 Structure	All text passages are included that mention the structure, composition and order of the tool's content.	*"Nicely structured and not so much kitsch, but really well structured and clear*
	2.2 Handling	All text passages are included that refer to the handling of the individual interactive elements of the tool and its navigation.	*"It's well known that there are further explanations to be found if you click on an underlined text passage."*
	2.3 Design	All text passages are included that refer to the design of the tool and its individual components, such as graphics, for instance.	*"Yes, as I said about the question marks. For me, it was always the case that I didn't take it all that seriously […]."*

Table S5. Continued

Main category	Subcategory	Definition	Anchor example
3. Comprehension<br><br>Comprehension includes statements concerning comprehension and perspectives of comprehensibility as a prerequisite for comprehension as well as statements about the difficulty of the test questions and exercises.	3.1 Comprehension of the content	All text passages are included in which it becomes clear how the participants can understand the contents.	*“What this means isn’t clear to me at the moment. No, I don’t understand it.”*
	3.2 Difficulty of the exercises	All passages are included that describe the perceived degree of difficulty of the exercises and test questions, including the task itself.	*“Yes, it can be solved. I would have to concentrate properly and pull myself together by entering the right thing.”*
	3.3 Text difficulties*	All passages are included in which statements about the readability, difficulty and understanding of a text passage are mentioned.	*“I think it is explained rather complicatedly. You have to read it several times in order to understand it.”*
	3.4 Transferability*	All passages are included that refer to the ability to apply what has been learned in practice and to be able to transfer it to other situations.	*“Yes, but I’m sure I can’t implement it yet.”*
4. Achievement of didactic objectives*		All text passages are included that allow conclusions to be drawn about the success or failure of the didactic strategy.	*“Well, it’s supposed to draw my attention to the fact that I’m probably wrong and that’s why I think it’s good.”*

* Inductively created categories
