# Supplementary material for: Development and Piloting of a Web-Based Tool to Teach Relative and Absolute Risk Reductions
Source: Int J Environ Res Public Health. 2022 Dec 1;19(23):16086. doi: 10.3390/ijerph192316086 (PMC9739880; doi:10.3390/ijerph192316086)
Supplement: Supplementary file 1 [file ijerph-19-16086-s001.zip › Table S4. Final concept of the tool.pdf]

Table S4. Final concept of the tool

Page	Goal	Content	Methodical strategy	Motivation strategies	Material
Home Page (HP)		• Objective and structure of the tool are explained		• Objectives of the tool are presented (C, S)	
Problem situation (PS1)	After completing the web-based tool, participants will be able to:<br>• Interpret risk reductions and describe the importance of correct interpretation	• Participants are faced with the problem of choosing from two price-reduced yoghurts the product with the largest absolute price reduction.	• Problem situation with an example from everyday life<br>• Interactive quiz followed by an explanation	• Awareness for the subject through a simple, plausible everyday problem (A, R, C)<br>• Illustrating the problem (A)<br>• Positive feedback (S)<br>• (Unknown) gaps in knowledge are revealed through the problem. (R; C)	• Illustration

(A) Attention; (R) Relevance; (C) Confidence; (S) Satisfaction

Table S6. Continued

Page	Goal	Content	Methodical strategy	Motivation strategies	Material
Problem situation (PS2)	• Interpret risk reductions and describe the importance of correct interpretation	• Transition from an everyday situation to a health-related situation<br>• Participants are asked to decide on one of two unspecified screening examinations.<br>• The same examination is shown with absolute and relative risk reduction.<br>• Solution follows after the decision.	• Problem situation with a health-related reference<br>• Interactive quiz followed by an explanation	• Transition from an everyday problem to a generally understandable medical problem (A, R)<br>• Working through the problem with interactive elements involving the learners; with positive feedback and solution (A; R; C)<br>• (Unknown) gaps in knowledge are revealed through the problem. (R; C)	• Illustration

(A) Attention; (R) Relevance; (C) Confidence; (S) Satisfaction

Table S6. Continued

Page	Goal	Content	Methodical strategy	Motivation strategies	Material
Initial situation (IS)	• Interpret risk reductions and describe the importance of correct interpretation	• Early detection examination is introduced as an example.<br>• Presentation of RCT findings	• Problem situation with health-related reference is continued.<br>• Pictogram shows procedure of an RCT and the event rates in the intervention and control groups.	• Generally understandable medical problem with relevance for the participants (A, R)<br>• Illustration (A)	• Pictogram
Absolute risk reduction (ARR)	• Interpret risk reductions and describe the importance of correct interpretation<br>• Calculate the absolute risk reduction	• Calculation of the absolute risk reduction using a pictogram<br>• Showing the formula for the calculation	• Problem situation with health-related reference is continued.<br>• Graphical presentation is supplemented by mathematical representation.	• Solutions are described verbally in a simplified way and presented using mathematical formulae for different requirements of the participants (R, C)<br>• Illustration (A)	• Pictogram<br>• Memory box

(A) Attention; (R) Relevance; (C) Confidence; (S) Satisfaction

Table S6. Continued

Page	Goal	Content	Methodical strategy	Motivation strategies	Material
Relative risk reduction (RRR)	• Interpret risk reductions and describe the importance of correct interpretation<br>• Calculate the relative risk reduction	• Calculation of the relative risk reduction using a pictogram<br>• Showing the formula for the calculation	• Problem situation with health-related reference is continued.<br>• Graphical presentation is supplemented by mathematical representation.	• Solutions are described verbally in a simplified way and presented using mathematical formulae for different requirements of the participants (R, C)<br>• Illustration (A)	• Pictogram<br>• Memory box

(A) Attention; (R) Relevance; (C) Confidence; (S) Satisfaction

Table S6. Continued

Page	Goal	Content	Methodical strategy	Motivation strategies	Material
Test your knowledge (TYK)	• Calculate the absolute risk reduction<br>• Calculate the relative risk reduction	• Checking knowledge on the calculation of absolute and relative risk reduction using an identically constructed fictitious case study.	• Fictitious problem with a reference group of 100 people for easy calculation is introduced.<br>• Possibility for checking and correction.	• Assistance can be optionally blended in (C, S)<br>• Possibility for checking and correction (S)	• Pictogram<br>• Calculation dialog box
Conclusion (C)	• Interpret risk reductions and describe the importance of correct interpretation	• Hints for the interpretation of the risk reduction are given.<br>• Links to pages for more in-depth information on early detection examinations and another example on the topic of nutrition.	• Summary in key words for a quick overview	• Translation of the imparted knowledge into short notes for use in own problem situations (R, C)	• Memory box

(A) Attention; (R) Relevance; (C) Confidence; (S) Satisfaction

Table S6. Continued

Page	Goal	Content	Methodical strategy	Motivation strategies	Material
Baseline risk (BS)	• Calculate the absolute risk reduction.<br>• Calculate the relative risk reduction.<br>• Interpret the impact of the baseline risk on absolute risk reduction by comparing the absolute risks at high and low baseline risks.	• Influence of the baseline risk on the absolute risk reduction using the example of statins for the prevention of a heart attack.<br>• Calculation of the absolute and relative risk reduction with high and low baseline risk and comparison of the absolute and relative risk reduction.	• Simplifying complex problem situations.<br>• Change in absolute risk reduction with different baseline risk is illustrated by own calculations.	• Assistance can be optionally blended in (C, S)<br>• Possibility for checking and correction (S)<br>• Illustration (A)	• Pictogram<br>• Calculation dialog box

(A) Attention; (R) Relevance; (C) Confidence; (S) Satisfaction

Table S6. Continued

Page	Goal	Content	Methodical strategy	Motivation strategies	Material
Additional exercises (AE)	• Calculate the absolute risk reduction.<br>• Calculate the relative risk reduction.<br>• Analyse the relative risk reduction of a real case study by calculating the event rates and deriving the absolute risk reduction from them.	• Three exercises with increasing stages of difficulty<br>• Exercise 1: Different group sizes and calculation of the event rates<br>• Exercise 2: Presentation in a table and introduction of the terms intervention group and control group.<br>• Exercise 3: Calculation of the absolute risk reduction based on a real case study.	• Difficulty of the exercises is increased stepwise<br>• Difficulties of the exercises are described in the selection.	• Exercise with a real example (A, R)<br>• Assistance can be optionally blended in (C, S)<br>• Possibility for checking and correction (S)<br>• The difficulty level is recognizable for the participants. (C, S)<br>• Illustration (A)	• Real case study<br>• Calculation dialog box

(A) Attention; (R) Relevance; (C) Confidence; (S) Satisfaction
