# Supplementary figures and images for: Development and Piloting of a Web-Based Tool to Teach Relative and Absolute Risk Reductions
Source: Int J Environ Res Public Health. 2022 Dec 1;19(23):16086. doi: 10.3390/ijerph192316086 (PMC9739880; doi:10.3390/ijerph192316086)

**Figure S1. Flow chart for the Internet search**

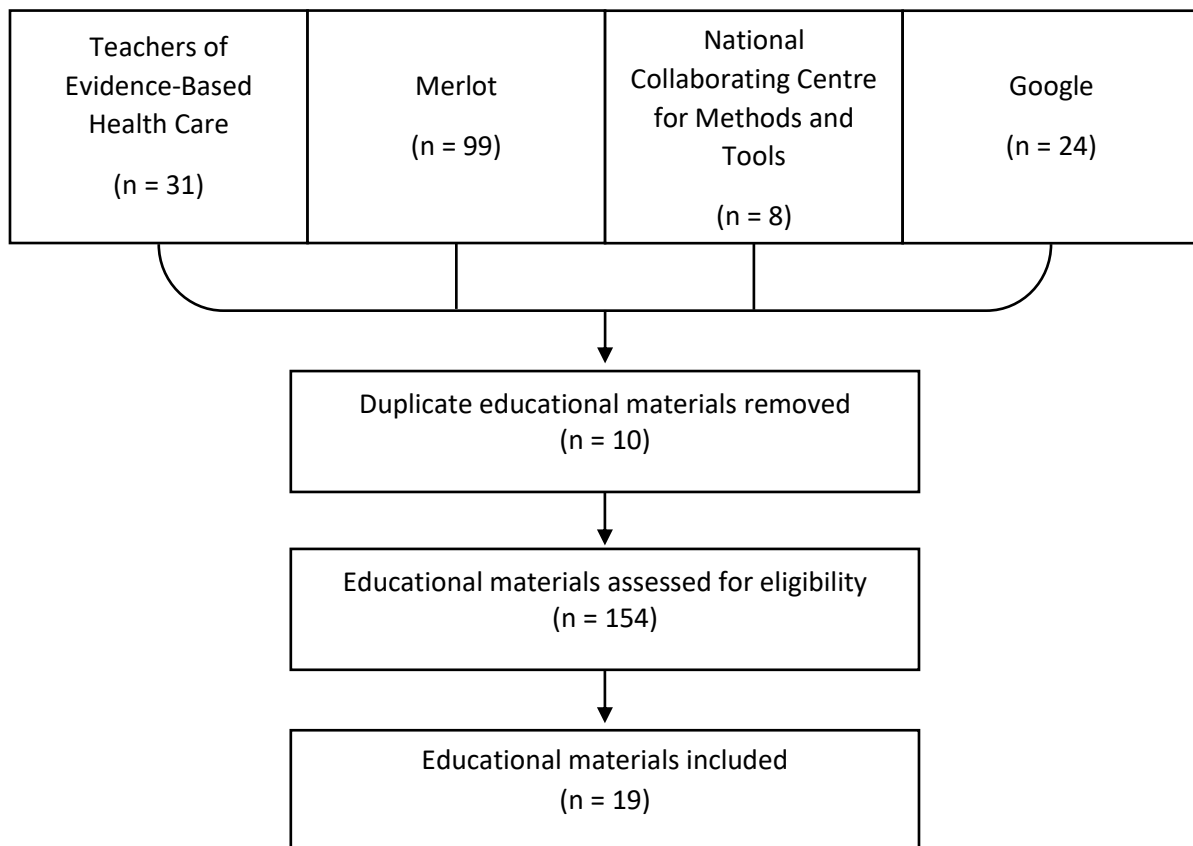

Supplement: Supplementary file 1 [file ijerph-19-16086-s001.zip › Figure S1. Flow chart for the Internet search.pdf]

**Figure S2. Flow chart for the systematic literature search**

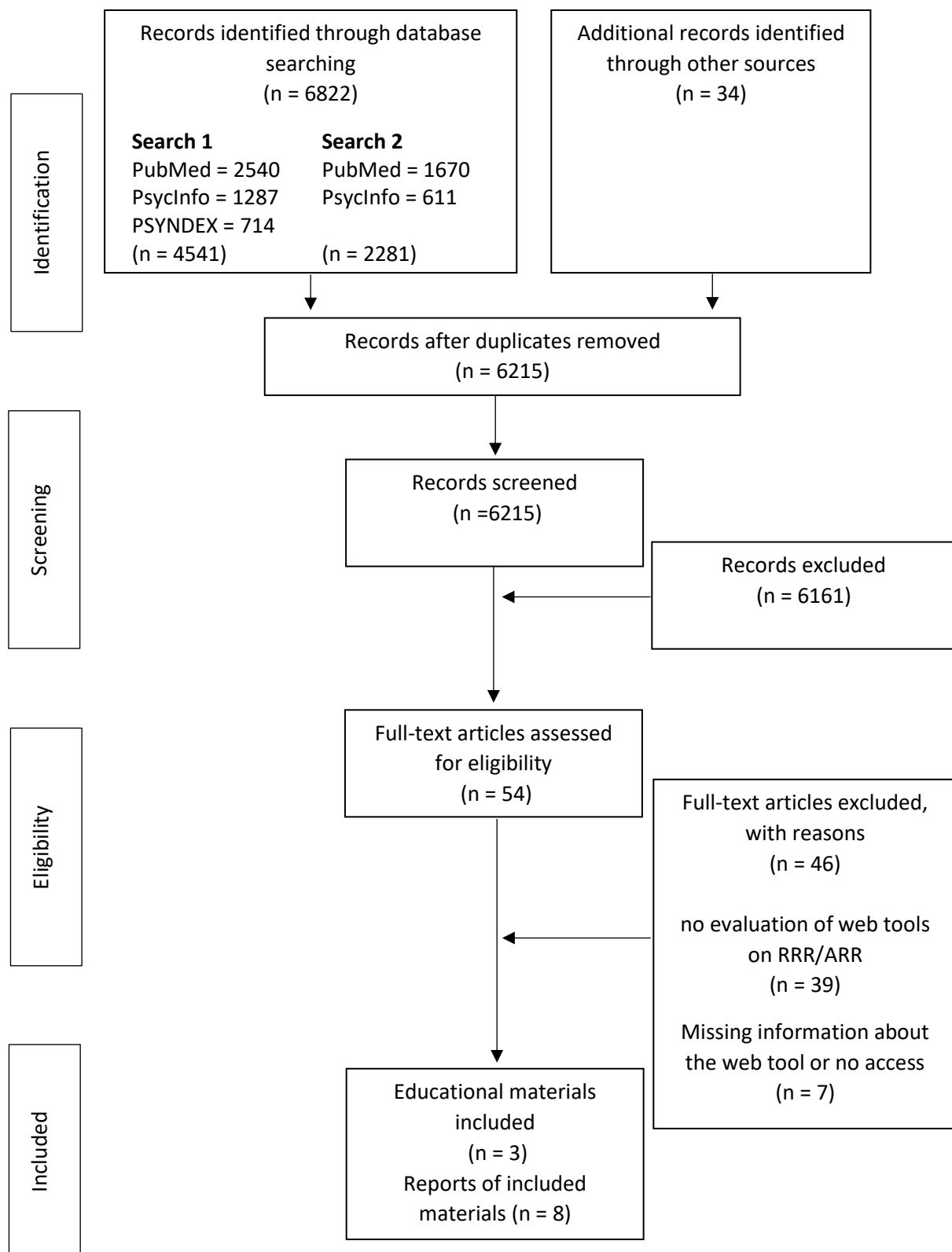

Supplement: Supplementary file 1 [file ijerph-19-16086-s001.zip › Figure S2. Flow chart for the systematic literature search.pdf]
